# Combat injury profiles among U.S. military personnel who survived serious wounds in Iraq and Afghanistan: A latent class analysis

**DOI:** 10.1371/journal.pone.0266588

**Published:** 2022-04-06

**Authors:** Edwin W. D’Souza, Andrew J. MacGregor, Amber L. Dougherty, Andrew S. Olson, Howard R. Champion, Michael R. Galarneau

**Affiliations:** 1 Medical Modeling, Simulation, and Mission Support Department, Naval Health Research Center, San Diego, California, United States of America; 2 Leidos, Inc., San Diego, California, United States of America; 3 Axiom Resource Management, Inc., San Diego, California, United States of America; 4 Uniformed Services University of the Health Sciences, Annapolis, Maryland, United States of America; 5 Section of Surgery, Uniformed Services University of the Health Sciences, Bethesda, Maryland, United States of America; John Hunter Hospital and University of Newcastle, AUSTRALIA

## Abstract

**Background:**

The U.S. military conflicts in Iraq and Afghanistan had the most casualties since Vietnam with more than 53,000 wounded in action. Novel injury mechanisms, such as improvised explosive devices, and higher rates of survivability compared with previous wars led to a new pattern of combat injuries. The purpose of the present study was to use latent class analysis (LCA) to identify combat injury profiles among U.S. military personnel who survived serious wounds.

**Methods:**

A total of 5,227 combat casualty events with an Injury Severity Score (ISS) of 9 or greater that occurred in Iraq and Afghanistan from December 2002 to July 2019 were identified from the Expeditionary Medical Encounter Database for analysis. The Barell Injury Diagnosis Matrix was used to classify injuries into binary variables by site and type of injury. LCA was employed to identify injury profiles that accounted for co-occurring injuries. Injury profiles were described and compared by demographic, operational, and injury-specific variables.

**Results:**

Seven injury profiles were identified and defined as: (1) open wounds (18.8%), (2) Type 1 traumatic brain injury (TBI)/facial injuries (14.2%), (3) disseminated injuries (6.8%), (4) Type 2 TBI (15.4%), (5) lower extremity injuries (19.8%), (6) burns (7.4%), and (7) chest and/or abdominal injuries (17.7%). Profiles differed by service branch, combat location, year of injury, injury mechanism, combat posture at the time of injury, and ISS.

**Conclusion:**

LCA identified seven distinct and interpretable injury profiles among U.S. military personnel who survived serious combat injuries in Iraq or Afghanistan. These findings may be of interest to military medical planners as resource needs are evaluated and projected for future conflicts, and medical professionals involved in the rehabilitation of wounded service members.

## Introduction

The U.S. military conflicts in Iraq and Afghanistan, including Operations Iraqi and Enduring Freedom (OIF/OEF), had the most combat casualties since Vietnam with more than 53,000 wounded in action [1]. The epidemiology of combat injuries in OIF/OEF varied from previous wars [2], as asymmetric warfare became more common [3–6] and the survivability of combat wounds increased [3, 7–9]. Blasts, often caused by improvised explosive devices, predominated the battlefield. As a result, blast-related traumatic brain injury (TBI) emerged as a preeminent wound of these conflicts [5, 10–12], and many casualties experienced polytrauma [4, 13, 14]. Case-fatality rates have sharply declined since World War II [3], as well as over the course of OIF/OEF [7, 9]. This change has been attributed to advances in personal protective equipment and field medicine [2, 3]. As more military personnel than ever are surviving combat wounds, clinical research efforts have prioritized long-term care and rehabilitation [15].

The novel epidemiology of combat injuries from OIF/OEF warrants further investigation. Previous studies have assessed injury patterns among specific samples of combat casualties, such as critically injured patients or those with certain types of injuries [13, 16–22]. To date, no study has examined wounding patterns, or injury profiles, among a large population of seriously wounded combat survivors. Information on injury profiles, including common co-occurring injuries, may inform military medical planners and leadership for future armed conflicts, and provide guidance for the knowledge, skills, and abilities required of military medical personnel.

Knowledge of combat casualty injury profiles could also inform further research, such as the evaluation of rehabilitation and recovery outcome metrics. Although measures such as the Injury Severity Score (ISS) have proven useful in predicting mortality [23–25], their utility in the assessment of long-term outcomes is unclear. One recent study found a pattern of postinjury multimorbidity (i.e., co-occurrence of two or more long-term health conditions) and poorer quality of life among military personnel with combat injury that was not associated with the highest levels of ISS [26]. This suggests that other factors, such as protracted impairments resulting from TBI or extremity trauma, may play a role beyond injury severity [11, 19].

The identification of combat casualty injury profiles may assist in refining patient management protocols to improve rehabilitation outcomes and overall well-being [27]. In addition, linking injury profiles to operational data may elucidate specific circumstances during wartime where certain combat injury profiles were more prevalent, and thus could influence future policies and prevention strategies. The objectives of the present study were to: (1) use latent class analysis (LCA) to identify injury profiles among U.S. military personnel who survived serious combat injuries; and (2) describe injury profiles by casualty and operational data.

## Methods

### Study population

Data for this study were obtained from the Expeditionary Medical Encounter Database (EMED), a deployment health repository at the Naval Health Research Center (NHRC), San Diego, California, that includes clinical records of U.S. service members injured during combat deployment. Clinical records were completed by providers in-theater and provided to NHRC where they were consolidated with patients’ medical records obtained from all levels of care. Patient records were retrospectively reviewed by certified nurse coders at NHRC and assigned *International Classification of Diseases*, *Ninth Revision*, *Clinical Modification* (ICD-9-CM) codes [28] and ISSs. Additional information on the EMED is available elsewhere [29]. The ISS is calculated as the sum of the squares of the highest Abbreviated Injury Scale [30] severity score in each of the three most severely injured body regions and quantifies overall injury severity for each casualty [23–25]. Only those with serious or greater injury severity (i.e., ISS ≥ 9) who survived their wounds through all levels of care were included. The study population included 5,227 casualty events that occurred during combat operations in Iraq and Afghanistan from December 2002 to July 2019. This study complied with all federal regulations governing the protection of human subjects in research and was approved by the Institutional Review Board (IRB) at NHRC. The approved IRB protocol (NHRC.2003.0025) issued a waiver of informed consent for this study.

### Variables

The Barell Injury Diagnosis Matrix, a two-dimensional table which categorizes injuries by body region (or site) and nature (or type) of injury, was used to classify injuries for each casualty [31]. In the Matrix, TBI is categorized as: Type 1 TBI (i.e., moderate-to-severe brain injury as indicated by an extended loss of consciousness and/or amnesia of the injury event); Type 2 TBI (i.e., mild brain injury as indicated by brief loss of consciousness or altered mental status); and Type 3 TBI (i.e., skull fracture without specification of intracranial injury). The Matrix has 36 rows that represent body regions and 12 columns that represent injury types. In this study, the “fractures” injury type was expanded to include “open” and “closed” fractures, which resulted in an additional column. Binary (1 or 0) injury variables were coded to indicate the presence or absence of the specific body region/injury type combination in each cell of the Matrix, and only populated cells in the Matrix were examined. Overall, 181 binary injury variables were derived for each combat casualty.

Other demographic, operational, and injury-specific variables were abstracted from the EMED for descriptive purposes. Demographic and operational variables included age at time of injury (18–24, 25–29, or 30+), sex, service branch (Army, Marine Corps, Navy, or Air Force), year of injury (2002–2008 or 2009–2019), and combat location (Iraq or Afghanistan). Injury-specific variables included injury mechanism (blast, gunshot wound, or other), combat posture at the time of injury (mounted [i.e., in a vehicle] or dismounted [i.e., on foot]), and ISS, which was categorized as serious (ISS 9–15), severe (ISS 16–24), and critical (ISS ≥ 25) [25].

### Statistical analysis

Analyses were performed using SAS software, version 9.4 (SAS Institute, Cary, North Carolina) and R software, version 3.6.2 (The R Project for Statistical Computing). The R package *poLCA* [32] was used for LCA, a probability model-based clustering algorithm [33, 34]. LCA was used to map the 181 binary injury manifest variables [34] onto classes termed “injury profiles.” Injury profiles represented mutually exclusive groups of combat casualties with commonly co-occurring injuries. Several LCA models were built, with the number of latent classes ranging from 1 to 10. The best model for each group was chosen using a combination of qualitative and quantitative measures, preferring models with more coherent classes, fewer parameters, and better fit statistics [34]. Binary injury manifest variables with a conditional item probability (i.e., class-specific indicator probability) of at least 0.30 were used to identify and label the classes in the LCA models. Interpretation of injury profiles was based on LCA results and input from subject matter experts. Fit statistics, including the Bayesian information criterion (BIC), sample-size adjusted BIC (SABIC), Akaike information criterion (AIC), and consistent AIC (CAIC), were computed [35]. Casualties were assigned to each class in the LCA model using the maximum-probability assignment rule. To evaluate the likelihood of misclassification, mean classification posterior probabilities were estimated for each class. Values above 0.70 indicated well-separated classes in the model [36], and entropy values greater than 0.80 indicated “good” model classification of individual cases into classes [37]. The selected LCA model yielded latent classes (or injury profiles) that were described by injuries with conditional item probabilities above the 0.30 threshold in each group. Chi-square tests assessed the distribution of demographic, operational, and injury-specific variables across injury profiles. Multiple hypothesis tests were conducted to compare the proportions of the levels of each categorical variable over all possible pair combinations across injury profiles. P-values were adjusted using the Holm method to control the family-wise error rate for multiple comparisons. An alpha level of 0.05 was used for statistical significance.

## Results

Table 1 summarizes fit statistics of the LCA models with classes ranging from 1 to 10. The BIC, SABIC, AIC, and CAIC statistics indicated the ideal model had between 6 and 10 classes. The 7-class LCA model was selected as the best model based on a combination of good fit (smallest CAIC statistic), model parsimony (fewer model parameters), and coherent, interpretable classes. The 7-class LCA model had an entropy statistic of 0.857 and outperformed most of the models in delineating the classes. Assignment of cases to unique classes using the maximum-probability assignment rule yielded mean class membership posterior probabilities above 0.90 for all seven classes (Table 2). Posterior probabilities of membership among classes where cases were not assigned did not exceed 0.05, indicating a very low expected misclassification rate.

**Table 1:** Fit statistics of latent class analysis models.

K	LL	BIC	SABIC	AIC	CAIC	Entropy
1	-121801.7	245153.1	244578.0	243965.5	245334.1	-
2	-117477.9	238063.7	236910.2	235681.9	238426.7	0.805
3	-114460.0	233586.1	231854.3	230010.1	234131.1	0.834
4	-111854.5	229933.2	227623.1	225163.0	230660.2	0.855
5	-110141.5	228065.6	225177.1	222101.1	228974.6	0.856
6	-108802.0	226944.7	223477.9	219786.0	228035.7	**0.859**
7	-107823.0	226544.9	222499.7	218192.0	**227817.9**	0.857
8	-107034.0	**226525.2**	221901.7	216978.1	227980.2	0.850
9	-106477.0	226969.2	221767.4	216227.9	228606.2	0.846
10	**-105908.3**	227390.1	**221609.9**	**215454.6**	229209.1	0.848

Bolded values indicate best fit for each respective statistic.

K, number of classes; LL, log likelihood; BIC, Bayesian information criterion; SABIC, sample-size adjusted BIC; AIC, Akaike information criterion; CAIC, consistent AIC.

**Table 2:** Classification posterior probabilities of 7-class latent class analysis model.

Class	1	2	3	4	5	6	7
1	**0.91**	0.04	0.02	0.01	0.02	0.02	0.02
2	0.03	**0.90**	0.01	0.02	0.00	0.01	0.01
3	0.01	0.00	**0.90**	0.02	0.00	0.00	0.00
4	0.01	0.03	0.05	**0.91**	0.02	0.00	0.01
5	0.03	0.01	0.01	0.03	**0.92**	0.00	0.03
6	0.01	0.00	0.00	0.00	0.00	**0.97**	0.00
7	0.02	0.01	0.01	0.01	0.03	0.00	**0.93**

Values in the table are probabilities of the most likely class membership (column) by the latent class membership assignment (row).

Bolded values indicate mean posterior probabilities.

Table 3 shows the injury types and sites with conditional item probabilities above the 0.30 threshold for each of the seven classes in the model. Injury profiles were defined as: open wounds (class 1); Type 1 TBI/facial injuries (class 2); disseminated injuries (class 3); Type 2 TBI (class 4); lower extremity injuries (class 5); burns (class 6); and chest and/or abdominal injuries (class 7). The distribution of the study population (N = 5,227) by injury profile was 18.8% (n = 981) in open wounds, 14.2% (n = 742) in Type 1 TBI/facial injuries, 6.8% (n = 353) in disseminated injuries, 15.4% (n = 804) in Type 2 TBI, 19.8% (n = 1,036) in lower extremity injuries, 7.4% (n = 387) in burns, and 17.7% (n = 924) in chest and/or abdominal injuries.

**Table 3:** Conditional item probabilities by injury type and site in the 7-class latent class analysis model.

Injury Type	Injury Site	Class 1	Class 2	Class 3	Class 4	Class 5	Class 6	Class 7
		(n = 981)	(n = 742)	(n = 353)	(n = 804)	(n = 1,036)	(n = 387)	(n = 924)
Internal organ	Type 1 TBI	0.035	**0.483**	0.275	0.041	0.010	0.053	0.011
Internal organ	Type 2 TBI	**0.355**	0.256	**0.362**	**0.835**	0.168	**0.312**	0.102
Internal organ	Chest	0.194	0.174	**0.459**	0.101	0.017	0.123	**0.385**
Internal organ	Abdomen	0.180	0.065	**0.489**	0.051	0.038	0.083	**0.312**
Open wounds	Face	**0.613**	**0.670**	**0.587**	**0.400**	0.113	**0.327**	0.073
Open wounds	Pelvis and urogenital	**0.531**	0.051	0.104	0.014	0.100	0.063	0.043
Open wounds	Shoulder and upper arm	0.292	**0.303**	0.078	0.027	0.075	0.091	0.145
Open wounds	Forearm and elbow	**0.476**	0.205	0.160	0.078	0.076	0.111	0.074
Open wounds	Wrist, hand, and fingers	**0.502**	0.178	0.124	0.053	0.069	0.101	0.057
Open wounds	Other and unspecified lower extremity	**0.791**	**0.390**	**0.452**	0.177	**0.516**	**0.347**	0.125
Contusion/superficial	Eye	0.182	**0.366**	0.142	0.080	0.012	0.253	0.002
Contusion/superficial	Head, face, and neck unspecified	0.206	0.234	0.277	**0.309**	0.054	0.177	0.028
Burns	Head, face, and neck unspecified	0.041	0.060	0.017	0.006	0.002	**0.597**	0.000
Burns	Wrist, hand, and fingers	0.021	0.016	0.013	0.000	0.000	**0.511**	0.000
Burns	Other/multiple	0.011	0.017	0.003	0.020	0.004	**0.324**	0.004
Burns	Unspecified	0.114	0.110	0.050	0.007	0.004	**0.986**	0.009
Fracture (closed)	Chest	0.011	0.027	**0.352**	0.077	0.001	0.034	0.031
Fracture (closed)	Pelvis and urogenital	0.046	0.000	**0.331**	0.029	0.003	0.021	0.005
Fracture (closed)	Lower leg and ankle	0.047	0.010	**0.400**	0.181	0.086	0.080	0.002
Fracture (closed)	Foot and toes	0.032	0.000	**0.302**	0.183	0.076	0.052	0.003
Fracture (open)	Face	0.078	**0.417**	0.125	0.060	0.010	0.047	0.007
Fracture (open)	Thoracic/dorsal VCI	0.013	0.008	**0.332**	0.155	0.003	0.034	0.051
Fracture (open)	Lumbar VCI	0.036	0.017	**0.584**	0.243	0.012	0.072	0.041
Fracture (open)	Sacrum coccyx VCI	0.048	0.003	**0.326**	0.034	0.004	0.014	0.033
Fracture (open)	Wrist, hand, and fingers	**0.394**	0.086	0.067	0.007	0.014	0.040	0.033
Fracture (open)	Lower leg and ankle	0.247	0.034	**0.339**	0.064	**0.482**	0.177	0.008

TBI, traumatic brain injury; VCI, vertebral column injury.

Class 1 = open wounds; class 2 = Type 1 TBI/facial injuries; class 3 = disseminated injuries; class 4 = Type 2 TBI; class 5 = lower extremity injuries; class 6 = burns; and class 7 = chest and/or abdominal injuries.

Bolded values indicate conditional item probabilities above threshold of 0.3. Only injury types and sites with probabilities above threshold are shown.

Characteristics of the study population by injury profile are shown in Table 4. The study population was predominantly male (98.3%), aged 18–24 years (55.9%), and in the Army (70.6%). The majority were injured while deployed in Iraq (50.2%) between 2002–2008 (53.3%). Most casualties were injured by blasts (75.9%) and sustained serious injuries (ISS 9–15; 59.3%). A slightly higher proportion of the study population was mounted than dismounted at the time of injury (42.2% vs. 39.5%). All variables except for sex and age differed significantly across the injury profiles (*p*s < 0.001). The burns profile (class 6) had the highest percentage of Army service members (77.5%), whereas the open wounds profile (class 1) had the most Marines (33.5%). Compared with all other profiles, burns had the highest proportions of personnel injured in Iraq (73.6%) between 2002–2008 (79.3%), and Type 2 TBI (class 4) had the highest proportions injured in Afghanistan (69.2%) between 2009–2019 (66.3%). Blasts were the predominant injury mechanism for all injury profiles except for the chest and/or abdominal injuries group (class 7), which had a significantly higher proportion of gunshot wounds. The open wounds and disseminated injuries profiles (classes 1 and 3) had the highest proportions of service members dismounted and mounted at the time of injury, respectively. The disseminated injuries profile also had the highest percentages of severe (35.7%) and critical (47.0%) injuries. Conversely, the lower extremity injuries profile (class 5) was the least severe, with the lowest proportions of severe (14.7%) and critical (1.8%) injuries.

**Table 4:** Characteristics of combat casualties by injury profile in 7-class latent class analysis model.

Variable	Total	Class 1	Class 2	Class 3	Class 4	Class 5	Class 6	Class 7	*p*
	(N = 5,227)	(n = 981)	(n = 742)	(n = 353)	(n = 804)	(n = 1,036)	(n = 387)	(n = 924)	
**Age (years), n (%)**									0.11
18–24	2,921 (55.9)	560 (57.1)	405 (54.6)	189 (53.5)	448 (55.7)	589 (56.9)	213 (55.0)	517 (56.0)	
25–29	1,285 (24.6)	267 (27.2)	177 (23.9)	87 (24.6)	196 (24.4)	255 (24.6)	87 (22.5)	216 (23.4)	
30+	1,021 (19.5)	154 (15.7)	160 (21.6)	77 (21.8)	160 (19.9)	192 (18.5)	87 (22.5)	191 (20.7)	
**Male, n (%)**	5,136 (98.3)	966 (98.5)	732 (98.7)	349 (98.9)	788 (98.0)	1,010 (97.5)	381 (98.4)	910 (98.5)	0.43
**Service branch, n (%)**									<0.001
Army	3,691 (70.6)	620 (63.2)^b,c,d,e,f^	532 (71.7)^a^	264 (74.8)^a^	607 (75.5)^a,g^	731 (70.6)^a^	300 (77.5)^a,g^	637 (68.9)^d,f^	
Marine Corps	1,371 (26.2)	329 (33.5)^b,c,d,e,f^	185 (24.9)^a^	82 (23.2)^a^	176 (21.9)^a^	268 (25.9)^a^	75 (19.4)^a,g^	256 (27.7)^f^	
Navy	109 (2.1)	21 (2.1)	15 (2.0)	4 (1.1)	15 (1.9)	24 (2.3)	8 (2.1)	22 (2.4)	
Air Force	56 (1.1)	11 (1.1)	10 (1.3)	3 (0.8)	6 (0.7)	13 (1.3)	4 (1.0)	9 (1.0)	
**Combat location, n (%)**									<0.001
Afghanistan	2,601 (49.8)	606 (61.8)^b,d,e,f,g^	279 (37.6)^a,c,d,f^	215 (60.9)^b,d,e,f,g^	556 (69.2)^a,b,c,e,f,g^	442 (42.7)^a,c,d,f^	102 (26.4)^a,b,c,d,e,g^	401 (43.4)^a,c,d,f^	
Iraq	2,626 (50.2)	375 (38.2)^b,d,e,f,g^	463 (62.4)^a,c,d,f^	138 (39.1)^b,d,e,f,g^	248 (30.8)^a,b,c,e,f,g^	594 (57.3)^a,c,d,f^	285 (73.6)^a,b,c,d,e,g^	523 (56.6)^a,c,d,f^	
**Year of injury, n (%)**									<0.001
2002–2008	2,788 (53.3)	393 (40.1)^b,d,e,f,g^	471 (63.5)^a,c,d,f^	157 (44.5)^b,d,e,f,g^	271 (33.7)^a,b,c,e,f,g^	640 (61.8)^a,c,d,f^	307 (79.3)^a,b,c,d,e,g^	549 (59.4)^a,c,d,f^	
2009–2019	2,439 (46.7)	588 (59.9)^b,d,e,f,g^	271 (36.5)^a,c,d,f^	196 (55.5)^b,d,e,f,g^	553 (66.3)^a,b,c,e,f,g^	396 (38.2)^a,c,d,f^	80 (20.7)^a,b,c,d,e,g^	375 (40.6)^a,c,d,f^	
**Injury mechanism, n (%)**									<0.001
Blast	3,966 (75.9)	914 (93.2)^b,e,g^	580 (78.2)^a,c,d,e,f,g^	328 (92.9)^b,e,g^	753 (93.7)^b,e,g^	707 (68.2)^a,b,c,d,f,g^	368 (95.1)^b,e,g^	316 (34.2)^a,b,c,d,e,f^	
Gunshot wound	1,074 (20.5)	48 (4.9)^b,e,f,g^	127 (17.1)^a,c,d,e,f,g^	7 (2.0)^b,e,g^	22 (2.7)^b,e,g^	295 (28.5)^a,b,c,d,f,g^	4 (1.0)^a,b,e,g^	571 (61.8)^a,b,c,d,e,f^	
Other	187 (3.6)	19 (1.9)	35 (4.7)	18 (5.1)	29 (3.6)	34 (3.3)	15 (3.9)	37 (4.0)	
**Combat posture, n (%)**									<0.001
Mounted	2,208 (42.2)	227 (23.1)^b,c,d,e,f,g^	265 (35.7)^a,c,d,f,g^	277 (78.5)^a,b,e,g^	602 (74.9)^a,b,e,g^	400 (38.6)^a,c,d,f,g^	285 (73.6)^a,b,e,g^	152 (16.5)^a,b,c,d,e,f^	
Dismounted	2,067 (39.5)	645 (65.7)^b,c,d,e,f,g^	277 (37.3)^a,c,d,f,g^	35 (9.9)^a,b,d,e,g^	130 (16.2)^a,b,c,e,g^	442 (42.7)^a,c,d,f,g^	44 (11.4)^a,b,e,g^	494 (53.5)^a,b,c,d,e,f^	
Unknown	952 (18.2)	109 (11.1)	200 (27.0)	41 (11.6)	72 (9.0)	194 (18.7)	58 (15.0)	278 (30.1)	
**ISS, n (%)**									<0.001
Serious (9–15)	3,097 (59.3)	419 (42.7)^c,d,e,g^	344 (46.4)^c,d,e,g^	61 (17.3)^a,b,d,e,f,g^	653 (81.2)^a,b,c,f,g^	865 (83.5)^a,b,c,f,g^	186 (48.1)^c,d,e,g^	569 (61.6)^a,b,c,d,e,f^	
Severe (16–24)	1,225 (23.4)	309 (31.5)^d,e,g^	209 (28.2)^d,e,g^	126 (35.7)^d,e,f,g^	126 (15.7)^a,b,c,f,g^	152 (14.7)^a,b,c,f,g^	101 (26.1)^c,d,e^	202 (21.9)^a,b,c,d,e^	
Critical (25+)	905 (17.3)	253 (25.8)^c,d,e,g^	189 (25.5)^c,d,e,g^	166 (47.0)^a,b,d,e,f,g^	25 (3.1)^a,b,c,f,g^	19 (1.8)^a,b,c,f,g^	100 (25.8)^c,d,e,g^	153 (16.6)^a,b,c,d,e,f^	

ISS, Injury Severity Score.

Class 1 = open wounds; class 2 = Type 1 TBI/facial injuries; class 3 = disseminated injuries; class 4 = Type 2 TBI; class 5 = lower extremity injuries; class 6 = burns; and class 7 = chest and/or abdominal injuries.

Statistically significant difference (*p* < 0.05) compared with ^a^ Class 1, ^b^ Class 2, ^c^ Class 3, ^d^ Class 4, ^e^ Class 5, ^f^ Class 6, ^g^ Class 7.

## Discussion

The U.S. military conflicts in Iraq and Afghanistan resulted in a new pattern of injuries among combat casualties. To our knowledge, the present study is the first to describe injury profiles among combat casualties using LCA. Seven injury profiles were identified and described by demographic, operational, and injury-specific data, which reflected different periods of the OIF/OEF conflicts and highlighted ubiquitous injury types, such as TBI and lower extremity injuries [11, 19]. The findings may be of interest to military medical planners who project the logistics, resources, and skilled providers required to treat combat casualties with serious injuries in future conflicts, and to medical professionals involved in injury rehabilitation, as many military personnel with combat injuries may require life-long care [27].

One of the profiles identified in the present study indicated a wide range of open wounds marked by a high proportion of service members dismounted at the time of injury who were primarily injured by blasts. This profile appears to be similar to “dismounted complex blast injury,” which is characterized in the literature by extensive open wounds, including amputations and pelvic/urogenital injuries [21, 22]. The circumstances surrounding dismounted complex blast injury typically involve military personnel on foot patrol when an explosive device is activated nearby [22]. Survivors of these type of injuries face quality of life concerns due to resulting disabilities, and optimal rehabilitation strategies are necessary [38]. Pelvic protection has been developed for U.S. military personnel and future research is needed to determine its utility in a combat environment [39].

In contrast to the open wounds profile, the group with disseminated injuries, including injuries to internal organs and fractures (both open and closed), had the highest proportion of service members mounted in a vehicle at the time of injury. Most service members in this profile were also injured by blasts. A unique aspect of this profile was fractures to the vertebral column, which has been identified in previous research on mounted casualties [40, 41]. Though enclosure within a vehicle affords some protection compared with dismounted personnel, certain characteristics of the injury incident can increase risk for serious injury, such as vehicle rollover, and high velocity displacement [41]. A key variable missing from the present analysis was the type of vehicle mounted at the time of injury, which can impact injury patterns [40]. Over the course of OIF/OEF, vehicles have been improved to maximize operational effectiveness and increase the amount of protective armor. Humvees were widely used during the early phases of these conflicts, but were later phased out in favor of Mine-Resistant Ambush-Protected vehicles [42]. In addition, position in the vehicle can affect injury patterns, as a previous study found that gunners (i.e., operators of the weapon on top of the vehicle) had a higher percentage of extremity wounds compared with drivers and passengers [41]. As such, a more detailed analysis of mounted injuries is required to further define risk factors and develop potential preventive strategies.

The identification of TBI-related profiles was not surprising given that TBI emerged as one of the signature wounds of OIF/OEF, with an estimated 1 in 5 service members with mild TBI [10, 11]. One TBI profile consisted primarily of Type 2 TBI, whereas the other predominately involved Type 1 TBI, which generally results in worse long-term outcomes than mild Type 2 TBI [43]. Of note in the present study, both TBI profiles occurred in the presence of other injuries (e.g., wounds to the face). A prior descriptive account of combat-related TBI found that a significant proportion of service members sustain concomitant injuries [10], and these other injuries can slow the course of TBI recovery [44]. Future military TBI research should address co-occurring injuries, potentially by using injury severity specific to the non-head region, such as the extracranial ISS used by Stulemeijer et al. [44]. Furthermore, efforts should continue to identify innovative methods for monitoring and mitigating TBI on the battlefield, including sensor technology and improvements in helmet design [10, 45].

There were other notable findings of interest. The chest and/or abdominal injury profile was the only profile where the proportion of service members with gunshot wounds significantly outnumbered those injured by blasts. In addition, this profile was isolated to injuries to the internal organs in the mid-section, with no other injuries meeting the probability threshold. Current personal protective equipment may offer protection, but certain variables not accounted for in this study may impact its effectiveness, including overall fit and bullet/shrapnel trajectory. Another profile was predominated by burns. Most service members in this group were injured between 2002–2008 and mounted at the time of injury, which could reflect the vehicle types used earlier in the conflicts as described previously. Finally, the lower extremity injury profile was not surprising, as these injuries frequently occurred during OIF/OEF [19]. This profile also had the lowest overall injury severity, which may be indicative of low-energy blast injuries, such as when an individual is a significant distance from a blast event, or the improvised explosive device is of a lower explosive weight [22].

The present study had several strengths. The EMED allowed for abstraction of medical and tactical information (e.g., injury mechanism, combat posture) from the point of injury, which is generally difficult to obtain in austere combat environments. Further, the Barell Injury Diagnosis Matrix is a standard injury classification method endorsed by the Centers for Disease Control and Prevention [46], and all casualty records were reviewed and validated by professional nurse coders to ensure accuracy. There are also limitations that warrant mention. The injury profiles from LCA were probability-based in contrast to other potentially more precise methodologies such as three-dimensional surface wound mapping [47]. The conditional item probability threshold of 0.30 was a subjective criterion and injuries not meeting this threshold could have contributed to the injury profile within each class. It is also important to note that the combat posture variable (i.e., mounted/dismounted status) had a large proportion of missing data. Further research of incident-related factors is needed and may require collaboration with other U.S. government agencies to obtain sensitive data (e.g., amount of explosive, distance from blast). Additional studies are warranted to explore injury profiles among casualties with minor injuries and those who died of wounds or were killed in action, as the focus of the present study was service members who survived serious injuries and findings may not generalize to these other groups.

## Conclusion

The present study used LCA to classify combat injury patterns among U.S. service members who survived serious wounds from OIF/OEF. Some of the injury profiles aligned with previous research that has identified dismounted complex blast injury, as well as preponderance of TBIs and lower extremity trauma during OIF/OEF. Combat posture at the time of injury was independently associated with various injury profiles, including the open wounds and disseminated injury groups, which requires further examination as these complex injury profiles impact long-term health outcomes. Additional research may be beneficial to identify injury-related sequelae and outlook for recovery. As modern warfare evolves and the U.S. military prepares for the next conflict, the identification and evaluation of combat injury patterns is paramount to medical planning and resource projections, and rehabilitation of wounded service members.
